# Diagnostic Evaluation of Pelvic Inflammatory Disease

**DOI:** 10.1155/S1064744994000384

**Published:** 1994

**Authors:** Jeffrey F. Peipert, David E. Soper

**Affiliations:** 1 Department of Obstetrics and Gynecology Women and lnfants' Hospital Brown University School of Medicine 101 Dudley Street Providence RI 02905 USA; 2 Department of Obstetrics and Gynecology Medical College of Virginia Richmond VA USA

## Abstract

Pelvic inflammatory disease (PID) is a serious public health and reproductive health problem in the United States. An early and accurate diagnosis of PID is extremely important for the effective management of the acute illness and for the prevention of long-term sequelae. The diagnosis of PID is difficult, with considerable numbers of false-positive and false-negative diagnoses. An abnormal vaginal discharge or evidence of lower genital tract infection is an important and predictive finding that is often underemphasized and overlooked. This paper reviews the clinical  diagnosis and supportive laboratory tests for the diagnosis of PID and outlines an appropriate diagnostic plan for the clinician and the researcher.


Infectious Diseases in Obstetrics and Gynecology 2:38–48 (1994)
© 1994 Wiley-Liss, Inc.

### Diagnostic Evaluation of Pelvic Inflammatory Disease

**Jeffrey F. Peipert and David E. Soper**

*Department of Obstetrics and Gynecology, Women and Infants' Hospital, Brown University School of Medicine, Providence, RI (J.F.P.), and Department of Obstetrics and Gynecology, Medical College of Virginia, Richmond, VA (D.E.S.)*

Abstract

**Pelvic inflammatory disease (PID) is a serious public health and reproductive health problem in the United States. An early and accurate diagnosis of PID is extremely important for the effective management of the acute illness and for the prevention of long-term sequelae. The diagnosis of PID is difficult, with considerable numbers of false-positive and false-negative diagnoses. An abnormal vaginal discharge or evidence of lower genital tract infection is an important and predictive finding that is often underemphasized and overlooked. This paper reviews the clinical diagnosis and supportive laboratory tests for the diagnosis of PID and outlines an appropriate diagnostic plan for the clinician and the researcher.** © 1994 Wiley-Liss, Inc.

Key words

Pelvic inflammatory disease, salpingitis, adnexitis

An early and accurate diagnosis of pelvic inflammatory disease (PID) is of paramount importance for the effective management of the acute illness and for the prevention of long-term sequelae such as infertility, ectopic pregnancy, chronic pelvic pain, and recurrent PID. The diagnosis of PID, however, is problematic as patients often present with minimal signs or symptoms and the severity of clinical signs varies widely. The changing etiologic, epidemiologic, and clinical profile of the disease magnifies this difficulty. Patients with PID may have minimal pain and atypical symptoms and remain undiagnosed, or they may be misdiagnosed as having gastrointestinal or noninfectious gynecologic illnesses.[1–5] Undiagnosed PID results in failure to treat or delay of treatment, which has been associated with an increased risk of long-term sequelae.[6–8]

### CASE ILLUSTRATION

A 20-year-old university undergraduate presented to a medical clinic for evaluation of burning with urination and abdominal discomfort. Symptoms of abdominal and pelvic discomfort and dysuria began 1–2 days prior to evaluation. The patient had never been pregnant, had no significant medical or surgical history or history of sexually transmitted diseases (STDs), used condoms for contraception, and was in a monogomous relationship for the past 2–3 years.

The practitioner evaluating the patient noted white blood cells (WBCs) in the clean-catch urinalysis and abdominal tenderness on examination. No pelvic examination or cultures were performed. The patient was diagnosed with a urinary tract infection and was told she might also have PID. She was given a prescription for ofloxacin, 300 mg b.i.d., which she filled and started immediately.

The patient followed-up with the student health clinic and was seen by a nurse practitioner 2 days later. On pelvic examination, she was noted to have abdominal, cervical motion, and adnexal tenderness. She was told that her examination was consistent with PID, and her prescription was changed to

Address correspondence/reprint requests to Dr. Jeffrey F. Peipert, Department of Obstetrics and Gynecology, Women and Infants' Hospital, 101 Dudley Street, Providence, RI 02905.

Received March 3, 1994

Accepted June 2, 1994

Review Article ofloxacin, 400 mg b.i.d., and metronidazole, 500 mg b.i.d., for 14 days. Instructions were given to have her sexual partner evaluated and treated.

Concerned with a 2-week course of antibiotic therapy and the implication of an STD, the patient sought consultation with a gynecologist, then 5 days after the onset of symptoms. By this time, her symptoms were almost resolved with the exception of mild vulvar pruritus. On examination at this evaluation, she had a cloudy-white discharge in the vaginal vault but no abdominal or pelvic tenderness. The vaginal pH was 4.2. Microscopic evaluation of the discharge revealed no WBCs, trichomonads, or clue cells; occasional pseudohyphae were noted. She was given a prescription for candidal vaginitis and was told to continue her course of antibiotics for "presumed" PID. The patient had numerous questions regarding her diagnosis, prognosis, and therapy.

This case illustrates the difficulties encountered in the routine diagnosis of PID. Many primary practitioners are reluctant to perform a pelvic examination. In addition, the importance of evaluating the vaginal discharge is illustrated in this example. Was the urine contaminated with excess vaginal WBCs secondary to a lower genital tract infection? Was the pyuria the result of inflammation of the bladder due to infection of the pelvic organs adjacent to the bladder? Or was the vaginal discharge normal and the patient had a case of urethritis and cystitis? A simple saline preparation of the vaginal discharge would add important diagnostic information.

### PROBLEMS WITH THE CLINICAL DIAGNOSIS OF PID

The clinical diagnosis of PID based on a bimanual pelvic examination and traditional laboratory testing has serious limitations.[9,10] Both overdiagnosis and underdiagnosis are quite common, and the list of conditions that must be considered in the differential diagnosis of PID is extensive (Table 1).

**TABLE 1. Differential diagnosis of PID**

| |
|---|
| Acute appendicitis |
| Ectopic pregnancy |
| Endometriosis |
| Ovarian cyst or tumor |
| Hemorrhage |
| Rupture |
| Torsion |
| Peritonitis |
| Pyelonephritis |
| Ruptured or perforated viscus |
| Intraperitoneal hemorrhage |
| Generalized sepsis |
| Urinary tract infection |
| Uterine myoma |
| Pelvic adhesions |
| Chronic salpingitis |
| Acute gastrointestinal inflammation |
| Miscellaneous |

Jacobson and Westrom[3] noted that one-third of women with the clinical diagnosis of PID have been misdiagnosed. Twelve percent were found to have other pathology such as appendicitis, endometriosis, hemorrhagic corpus luteum, or ectopic pregnancy; 23% were normal upon laparoscopic evaluation.[3] Laparoscopic evaluation of patients with other clinical diagnoses can also reveal unsuspected PID.[3] By using more stringent criteria for the diagnosis, such as the presence of fever or leukocytosis, the specificity of the diagnosis can be increased. However, this increase in specificity will result in a marked reduction in sensitivity.[10,11]

The limitations of the clinical diagnosis of PID underscore the need for more accurate diagnostic techniques that are widely available and cost-effective.[12] What follows is a critical review of the current techniques used in the clinical diagnosis of PID, including historical risk factors, physical examination, laboratory testing, and more elaborate tests such as endometrial biopsy, endovaginal ultrasonography, and laparoscopy. As these data are reviewed, the reader should consider how the accurate diagnosis of PID can be made with concern for both patient and health-care costs.

### HISTORIC RISK FACTORS AND RISK MARKERS

From case-control studies in the United States of women with confirmed PID, the patients with PID were younger, more often nonwhite, single, unemployed, had less education, consumed more alcohol, were more likely to use tobacco, and were more likely to douche than a sexually active control group without PID.[13,14] They also initiated sexual activity earlier, reported a higher number of lifetime sexual partners, had intercourse more frequently, had frequent change of sexual partners, and reported a history of *Neisseria gonorrhoeae*, *Chlamydia trachomatis*, or PID more often than did controls. In addition, cases with proven PID were less likely than control subjects to use any birth-control method.[13]

Health-care behavior impacts on the risk of acquiring PID. The risk of an upper genital tract infection increases when there is late medical consultation for the diagnosis and treatment of STDs, when there is noncompliance with medical therapy, and when the sexual partner is not treated.[13]

Contraceptive history influences the occurrence of PID. Studies have shown that barrier methods may decrease the risk of PID, presumably by protecting against cervical infection.[15] The intrauterine device (IUD) may be associated with an increased risk of PID, but his risk appears to be limited to the 1st several months after IUD insertion.[16–18] However, women at low risk of acquiring an STD have little increased risk of PID with IUD use.[19] A tubal ligation markedly decreases the risk of ascending infection from the lower genital tract.[20] The literature is unclear with regard to the effect of oral contraceptives on the risk of PID. It would appear that, while the use of oral contraceptives increases a woman's risk for chlamydial endocervical infection, it decreases the risk of developing overt PID. In addition, women using oral contraceptives with laparoscopically proven salpingitis tend to have a milder stage of disease. It is unclear whether or not the oral contraceptives prevent "silent salpingitis." Numerous human and animal studies exist,[21–29] yet the impact of oral contraceptives on PID remains unclear due to numerous confounding behavioral variables in this complex relationship. Additional studies are needed to sort out this association.

While epidemiologic risk factors and risk markers may be helpful in determining who is at an increased risk of PID, these factors may play only a small role in the diagnosis of an individual patient. In general, women at risk for contracting an STD are at risk to develop PID.

### SYMPTOMS

Numerous symptoms are associated with upper genital tract infection. Pelvic pain is one of the most common complaints encountered in gynecology and may represent PID. Pain associated with PID is most often described as dull, continuous, low abdominal or bilateral pelvic in location, and gradual in onset. In gonococcal PID, the pain is often more intense, and medical consultation is sought usually within a few days. In contrast, chlamydial PID is less intense, and the patient may wait a week or longer prior to seeking medical attention.[30] In "asymptomatic salpingitis," or "atypical PID," pain may not be present.

Symptoms typically occur during menstruation or the proliferative phase, rather than the luteal phase of the cycle.[9,31] Abnormal uterine bleeding, including intermenstrual bleeding and menorrhagia, is correlated with histologic evidence of endometritis.[32] These symptoms were reported by one-third of laparoscopically proven cases of acute salpingitis.[33] Menorrhagic or intermenstrual bleeding is more common in chlamydial PID than in cases of gonorrhea-associated or non-STD-associated PID.[30]

Among cases of women with proven salpingitis who recorded their temperature prior to consulting medical care, 40% reported febrile illness.[9] Chills were reported by 12%.[33] A vaginal discharge has been considered a hallmark of PID.[30] Three-fourths of women with laparoscopically verified PID gave a history of an abnormal vaginal discharge, and all women with salpingitis had an abnormal discharge on physical examination.[33,34] Urinary symptoms, such as frequency or a burning sensation, were present in 15–20% of laparoscopically verified PID.[9] Gastrointestinal symptoms are uncommon in mild or moderate salpingitis, but nausea and vomiting are common complaints in severe PID.[30] Symptoms of proctitis are infrequent; if present, these symptoms should raise a suspicion of a cul-de-sac abscess or gonoccoccal proctitis.[9] The frequency of various symptoms in cases of laparoscopically proven PID is presented in Table 2.

**TABLE 2. Prevalence of symptoms in cases of laparoscopically proven PID**

| Symptom | Percent |
|---|---|
| Abnormal vaginal discharge | 55–75 |
| Reported elevated temperature | 35–45 |
| Irregular bleeding | 30–35 |
| Urinary symptoms | 15–20 |
| Chills | 10–15 |
| Nausea and vomiting | 5–10 |
| Anorectal symptoms | 5–10 |
| Right upper-quadrant pain | 5 |

In summary, numerous genitourinary tract symptoms occur in patients with PID. Pain may or may not be present. Since no symptom has significant specificity or sensitivity with respect to the diagnosis of PID, patients with any genitourinary symptoms should be considered candidates for the diagnosis.

### PHYSICAL EXAMINATION

Hagdu et al.[35] used multivariate logistic regression analysis to identify important predictors of PID. The most important predictor of PID was found to be an abnormal vaginal discharge which was found in virtually all women with salpingitis. The importance of a careful assessment for mucopurulent cervicitis and cervical inflammation and an evaluation of the vaginal discharge cannot be overstated.

Bacterial vaginosis also has been found to be an independent risk factor for PID. In a study of women with suspected PID, bacterial vaginosis was found in 9/31 women with proven PID and 0/14 without PID.[36] In a recent study of the microbiologic etiology of acute salpingitis, an abnormal vaginal flora characterized by bacterial vaginosis or its intermediate state was almost always present in patients with acute salpingitis. The authors noted that, other than *N. gonorrhoeae* and *C. trachomatis*, bacterial vaginosis microorganisms were the most common group of microorganisms isolated from the upper genital tract.[37] In contrast, Faro and colleagues[38] did not find this association in their study of 41 women with clinically severe PID.

Leukorrhea is present when inflammatory cells [polymorphonuclear (PMN) leukocytes] are the predominant cells noted during microscopy of a vaginal saline preparation. Microscopic evaluation of the vaginal secretion is a simple and rapid test that can provide valuable information in the diagnosis of PID.[35] The proportion of inflammatory cells indicates the degree of host response. A normal wet mount of the vaginal secretions, plus clear cervical mucus, is felt to reliably exclude upper genital tract infection.[30] The presence of a mucopurulent cervical discharge is defined as endocervical mucopus or the presence of $\geq 10$ PMN leukocytes per high power field ($\times 1,000$) in Gram-stained endocervical smears.[39] The presence of leukorrhea or mucopus is consistent with the diagnosis of lower genital tract infection. Leukorrhea or mucopurulent cervicitis is suggestive of PID in women with coexisting pelvic pain or pelvic organ tenderness.[39,40]

**TABLE 3. Sensitivities and specificities of selected physical examination findings**

| Sign | Sensitivity (%) | Specificity (%) |
|---|---|---|
| Adnexal tenderness[12] | 95 | 74 |
| Bilateral tenderness[12] | 58 | 92 |
| Cervical motion tenderness[12] | 82 | 72 |
| Palpable mass[12] | 48 | 75 |
| Abdominal guarding[14] | 64 | 63 |
| Clinical cervicitis[41] | 75 | 93 |

Kahn and colleagues[12] evaluated data on the diagnosis of PID and combined data from 14 studies for review. Adnexal tenderness had a high sensitivity (95%), but a low specificity (74%) in predicting PID. Other sensitivities and specificities of physical examination findings based on this review[12] and a recent study from a primary care setting[41] are presented in Table 3.

Since the treatment of a tubo-ovarian abscess (TOA) requires hospitalization, the presence of a palpable adnexal mass is an important clinical finding. The clinical diagnosis of a TOA is based on clinical criteria for PID in conjunction with a palpable adnexal complex. The predictive value of a palpable mass, however, is low as this finding was reported by experienced examiners in 25% of cases with normal findings at laparoscopy.[9] Ultrasound may be useful to further characterize the mass. Other signs, symptoms, and laboratory tests are not helpful in the diagnosis of a TOA.

### LABORATORY TESTING

There is no one diagnostic test that is pathognomonic for PID. Several new tests have been proposed in the evaluation and diagnosis of women with suspected upper genital tract infection. These tests include C-reactive protein (CRP),[30,42–45] endometrial biopsy,[32,36,45–47] and endovaginal ultrasound.[48–52] It is clearly important to develop and to carefully evaluate these and other diagnostic tests that would improve and facilitate the diagnosis of PID. For the sake of brevity, only a few of the more predictive laboratory tests will be reviewed.

In 4 of 4 studies reviewed by Kahn et al.,[12] an elevated CRP was found to be a significant predictor. Sensitivities ranged from 74 to 93%, and specificities ranged from 50 to 90%.[30,42–45] In addition, levels of CRP appear to reflect the severity of laparoscopically proven salpingitis. The ability of a microbial substance to stimulate the synthesis of hepatic acute-phase proteins such as CRP is a prominent pathophysiologic mechanism of PID.[30]

Westrom[33] noted an elevated erythrocyte sedimentation rate (ESR) >15 mm/h in 76% of patients with visually diagnosed acute salpingitis, but also in 53% of women with suspected salpingitis but normal findings at laparoscopy. A significant association was noted between the ESR and the stage of salpingitis at laparoscopy and with chlamydia-associated salpingitis. When comparing ESR and CRP, Wasserheit et al.[43] found that an elevated CRP was a more sensitive and specific predictor of PID than was ESR.

Despite widespread belief by clinicians, an elevated WBC count is not a reliable predictor of PID, nor does it correlate with the severity of tubal inflammation or need for hospitalization. In fact, fewer than 60% of women with a laparoscopic diagnosis of acute salpingitis have a WBC count >10,000 cells/ml.[9]

Even though no one diagnostic test is pathognomonic for PID, it would appear that CRP has sufficient predictive value to be utilized in the diagnosis of PID. Additional studies are necessary to determine if CRP remains predictive in cases of mild and atypical PID.

### MICROBIOLOGIC TESTING

*N. gonorrhoeae* has been called the "classic" PID-causing agent.[9] In studies from the United States and Canada, *N. gonorrhoeae* has been isolated from the lower genital tract in 44–70% of women with acute PID, depending on diagnostic inclusion criteria.[9] Isolation of gonococci from the upper genital tract of patients with PID is usually between 10 and 54%.[37,53,54] Based on culture or serologic evidence, infection with *C. trachomatis* may be present in up to 38%[55] of hospitalized cases and 52% of women treated as outpatients for PID.[56,57]

Positive tests for *N. gonorrhoeae* or *C. trachomatis* also have been found to be significant predictors of PID in 2 well-designed studies.[35,43] Studies evaluating the identification of microorganisms and the diagnosis of PID are often limited by the microbiologic technique used for identification. While culture has traditionally been the gold standard for the diagnosis of both gonorrhea and chlamydia, recent studies have shown that the polymerase chain reaction (PCR) assay may be a more sensitive method.[58,59] PCR is not only sensitive, but is highly specific. In a study evaluating 15 women with laparoscopically verified salpingitis, Witkin et al.[60] found 60% (9/15) had positive PCR testing for chlamydia. Only 2 of these positive results had positive cervical cultures. The authors[60] concluded that PCR is a more sensitive and more rapid test for *C. trachomatis* in this population of women with acute salpingitis.

Evidence of lower genital tract infection, either by microbiologic testing or by evaluation of cervical Gram stain or vaginal discharge, is a hallmark of upper genital tract infection. In fact, the clinical diagnosis of PID is suspect in women who do not test positive for *N. gonorrhoeae* or *C. trachomatis* or who fail to show evidence of lower genital tract inflammation.

### ENDOMETRIAL BIOPSY

An endometrial biopsy can be a useful alternative to laparoscopy as an objective test in the diagnosis of PID.[32,36,46,47] It is a simple office or outpatient procedure that can be performed with or without a paracervical anesthetic. Endometrial sampling can be obtained with a variety of devices. A Novak curette, a VABRA (Berkeley Medevices, Inc., Berkeley, CA) endometrial suction curette, or a flexible endometrial sampling device such as the Pipelle (Unimar, Inc., Danbury, CT) or Gynosampler (Gynopharma, Inc., Somerville, NJ) can be used. The histopathologic diagnosis is usually available in 3–4 days. If an endometrial biopsy is used for histologic evaluation and culture of the upper genital tract in patients with suspected upper genital tract infection, antibiotics should be administered immediately after the sample is obtained.

The histologic features of the endometrium that are associated with infection of the upper genital tract and laparoscopically proven salpingitis include the presence of PMN leukocytes in the endometrial surface epithelium, dense subepithelial stromal lymphocytic infiltration, germinal centers containing transformed lymphocytes, and plasma cells in the endometrial stroma.[41,55,61]

Recent studies have shown a fair correlation between endometritis as demonstrated histologically and the laparoscopic diagnosis of PID.[36,47] Endometrial inflammation on biopsy has a good sensitivity (70–92%) and specificity (67–89%) for the diagnosis of PID.[43,44,55] Using a definition of histologic endometritis of >1 plasma cell/×120 field in the endometrial stroma plus >5 PMN leukocytes/×400 field in the endometrial surface gave the best prediction of laparoscopically proven salpingitis. This combination of histologic findings gave a sensitivity of 92% and a specificity of 87%.[61] Sellors and colleagues[41] demonstrated that an endometrial biopsy was more than 90% specific in predicting histopathologic evidence of salpingitis. Additional prospective studies are necessary in women with classic PID (with pain) and atypical PID (without pain) to confirm the predictive values of histopathologic criteria for endometritis in diagnosing patients with suspected upper genital tract infection.

An endometrial biopsy is also an indispensable tool in evaluating the endometrium of patients undergoing laparoscopy. If laparoscopic findings are not consistent with visual criteria for salpingitis, acute or chronic endometritis may be the source of the patient's symptoms.

An endometrial evaluation with outpatient sampling devices is an underutilized procedure in the diagnosis of upper genital tract infection. Histologic evidence of endometritis confirms the presence of upper genital tract inflammation and supports the diagnosis of PID.

### ULTRASONOGRAPHY

Transabdominal ultrasonography is useful in identifying complicated PID, e.g., TOA or inflammatory complex, but has not been very effective in distinguishing uncomplicated PID from other gynecologic conditions such as endometriosis or ovarian cysts.[62–64] The use of endovaginal probes has refined the imaging of the pelvic organs.[48,49] Transvaginal ultrasonography is a relatively noninvasive diagnostic modality that can be combined with the pelvic examination for evaluating gynecologic disorders, including PID.[50,51]

In a recent prospective study of 51 women with suspected PID, Cacciatore et al.[52] demonstrated that a transvaginal sonogram suggestive of PID, e.g., thickened, fluid-filled tubes, had a sensitivity of 85% and a specificity of 100% for the diagnosis of plasma cell endometritis as determined by an endometrial biopsy. The positive and negative predictive values for the group were 100 and 95%, respectively. The authors[52] concluded that transvaginal sonography can aid in the outpatient evaluation of women with suspected PID.

The findings of Cacciatore and colleagues[52] should be validated in other cohorts of women with both mild and severe cases of PID. As clinicians gain more experience with transvaginal sonography, this technique may become useful in evaluating patients with the presumptive diagnosis of PID.

### CLINICAL CRITERIA FOR PID

Clinical criteria for the diagnosis of PID have been outlined on several occasions[65–68] and are listed in Table 4. The Centers for Disease Control and Prevention (CDC) has released revised clinical criteria for PID which are summarized in Table 5.[68]

Jacobson and Westrom[3] evaluated the accuracy of conventional signs and symptoms of PID as assessed by visual confirmation of acute salpingitis. Diagnostic accuracy could be improved by increasing the number of positive parameters. They also noted that a marked increase in the number of inflammatory cells in the wet smear of vaginal secretions was found in women with PID. This test could be used to exclude the possibility of PID in women with abdominal pain.

Criteria endorsed by the Infectious Disease Society for Obstetrics and Gynecology (IDSOG) have been outlined by Hager et al.[65] The IDSOG guidelines eliminate the previously required chief complaint of abdominal pain. This is an important improvement if we are interested in patients with atypical PID who may not have abdominal pain.

Soper[67] proposed a set of clinical criteria that was a compromise between the 2 previously published guidelines. In these guidelines, the importance of signs of lower genital tract infection is stressed. In addition, the endometrial biopsy is proposed as an adjunct to confirm the clinician's suspicion of upper genital tract infection.

The CDC's minimum clinical criteria for PID include abdominal tenderness, cervical motion tenderness, and adnexal tenderness.[68] Based on the presence of all 3 of these findings, empiric treatment of PID should be instituted in the absence of an established cause other than PID. The additional criteria listed are proposed for patients with severe clinical signs in whom incorrect diagnosis and management may cause unnecessary morbidity.

Unfortunately, the clinical criteria proposed have

TABLE 4. Clinical criteria for the diagnosis of PID

| Hager et al.[65] | Sweet[66] | Soper[67] |
|---|---|---|
| | *Major Criteria (All Must Be Present)* | |
| Abdominal tenderness<br>Cervical motion tenderness<br>Adnexal tenderness | Abdominal tenderness<br>Cervical motion tenderness<br>Adnexal tenderness | Adnexal tenderness<br>Signs of lower genital tract infection |
| | *Minor Criteria (Additional Criteria Increase the Specificity of the Diagnosis)* | |
| Endocervical Gram stain positive for *N. gonorrhoeae* | >5 WBCs/×1000 fieldgram on gram stain of endocervix | Histologic evidence of endometritis |
| Temperature >38°C | Temperature >38°C | Elevated CRP or ESR |
| Leukocytosis (>10,000) | Leukocytosis (>10,500) | Temperature >38°C |
| Purulent material by culdocentesis or laparoscopy | Purulent material by culdocentesis | Leukocytosis (>10,000) |
| Pelvic abscess or inflammatory complex on bimanual examination or sonography | Pelvic complex by examination or sonography | Positive test for *C. trachomatis* or *N. gonorrhoeae* |
| | Evidence of *N. gonorrhoeae* or *C. trachomatis* in endocervix (+Gram stain for *N. gonorrhoeae* or monoclonal antibody for *C. trachomatis*) | |

TABLE 5. Revised CDC criteria for the diagnosis of PID

| |
|---|
| Minimum |
| Lower abdominal tenderness |
| Adnexal tenderness |
| Cervical motion tenderness |
| Additional |
| Routine |
| Oral temperature >38.8°C |
| Abnormal cervical or vaginal discharge |
| Elevated ESR |
| Elevated CRP |
| Laboratory documentation of cervical infection with *N. gonorrhoeae* or *C. trachomatis* |
| Elaborate |
| Histopathologic evidence of endometritis on endometrial biopsy |
| TOA on sonography or other radiologic tests |
| Laparoscopic abnormalities consistent with PID |

never been adequately validated in large prospective studies.[12,68] When attempts have been made to validate clinical criteria, the imprecision of clinical diagnosis is clear. Approximately one-third of women diagnosed with PID do not have PID when laparoscopic inspection is performed.[3]

### LAPAROSCOPY

It is believed that PID is best diagnosed visually by laparoscopic evaluation of the pelvic organs.[3,69,70] However, a diagnostic laparoscopy is expensive and is not always readily available. In addition, many women without visible salpingitis may have infection either within the tubal lumen or the endometrium and therefore may have a false-negative laparoscopy.[20,37] At the time of diagnostic laparoscopy, it is recommended that an endometrial biopsy be performed to rule out endometritis that has not progressed to acute salpingitis.

Three laparoscopic findings have been used as the minimal criteria for the visual confirmation of acute salpingitis: pronounced hyperemia of the tubal surface; edema of the tubal wall; and sticky exudate on the tubal surface and from the fimbriated ends of the fallopian tubes when patent.[3] The severity of disease at laparoscopic evaluation correlates with the development of long-term sequelae.

### ATYPICAL PID

The classic picture of acute PID with abdominal, cervical motion, and abdominal tenderness may represent a small proportion of the entire spectrum of cases of PID. In fact, a growing body of evidence suggests that acute PID with lower abdominal pain as the chief complaint may represent the "tip of the iceberg" and that large numbers of women with relatively or completely painless upper genital tract infections are undiagnosed. The majority of women with tubal factor infertility have no history of signs or symptoms of PID.[71–73] Wolner-Hanssen et al.[1] have estimated that there may be 4–6 cases of atypical PID for every case of classic or typical PID.

These authors suggested that additional studies are necessary to determine the prevalence of histopathologic endometritis in women with mucopurulent cervicitis and in women with menorrhagia.

Although PID is believed to be associated with abdominal pain of recent onset, symptoms from other organ systems may also be due to upper genital tract infection. Genital tract symptoms (mucopurulent cervicitis, vaginal discharge, abnormal uterine bleeding, dysmenorrhea, or dyspareunia), gastrointestinal symptoms (nausea, vomiting, or proctitis), or urinary tract symptoms (dysuria, urgency, or frequency) may all be associated with PID.[1]

Most PID studies address only the classic presentations of the disease and fail to evaluate women with more atypical forms of the disease. Cates et al.[74] have attempted to identify predictors of atypical PID and found that women with atypical PID were demographically more like fertile control subjects than were women with overt PID. Behavioral characteristics of the atypical PID group were midway between those of the overt PID group and the fertile control group. The authors[74] concluded that the clinical predictors of atypical PID remain elusive. Clinicians should consider PID in the differential diagnosis of women with mucopurulent cervicitis and abdominal pain and in patients with unexplained uterine bleeding, dyspareunia, or atypical pelvic pain. Future prospective studies should address women with atypical as well as typical presentations of upper genital tract infection.

### DIAGNOSTIC APPROACH

For the clinician, the diagnosis of PID should be considered in any woman of reproductive age who has any genitourinary symptoms.[10] Clinicians should have a high index of suspicion and a low threshold for initiating treatment because the potential for serious long-term reproductive sequelae is great, even if the patient's symptoms are mild.

A comprehensive diagnostic evaluation will include a careful history to illicit risk factors for PID, as well as a pelvic examination, and careful assessment for lower genital tract infection, including tests for *N. gonorrhoeae* and *C. trachomatis*, testing for mucopurulent cervicitis, and evaluation of the vaginal wet preparation for leukocytes. Additional laboratory tests that may be helpful include ESR, CRP, and endometrial biopsy.

**TABLE 6. Effect of adjunctive clinical criteria on the specificity of the clinical diagnosis of PID[a]**

| Criteria | Specificity (%) |
|---|---|
| Major | |
| Lower abdominal pain | |
| Signs of lower genital tract infection | |
| Bilateral adnexal tenderness | |
| Minor | |
| Fever | |
| Palpable adnexal swelling | |
| Leukocytosis | |
| Elevated ESR or CRP | |
| Positive test for *N. gonorrhoeae* or *C. trachomatis* | |
| Major criteria (all three) | 61 |
| Major + one minor | 78 |
| Major + two minor | 90 |
| Major + three minor | 96 |

[a]Adapted from Mardh and Westrom.

The CDC suggests that the minimal criteria needed for the diagnosis of PID are abdominal, cervical motion, and adnexal tenderness.[68] The sensitivity and specificity of these criteria are unknown at this time and await further validation. It is imperative to evaluate the lower genital tract for evidence of infection. We believe that evidence of any pelvic organ tenderness and the presence of lower genital tract inflammation can be used to make the diagnosis of PID. If there is no evidence of lower genital tract infection (normal cervical mucus and no predominance of WBCs in the vaginal wet preparation), the likelihood of PID is quite low.

The likelihood of PID increases as the number of positive findings increase. Adjunctive criteria for the diagnosis of PID include elevated temperature, palpation of an adnexal complex, leukocytosis, elevated ESR or CRP, purulent material obtained by culdocentesis, and/or a positive test for a lower genital tract infection with *N. gonorrhoeae* or *C. trachomatis*. The specificity of the clinical diagnosis of PID increases to greater than 90% when 2 or more adjunctive criteria are present with findings of pain, adnexal tenderness, and leukorrhea (Table 6). More elaborate indicators (endometrial biopsy or laparoscopy) should be employed when the diagnosis is uncertain, when symptoms are severe and misdiagnosis may result in serious morbidity, or when the diagnosis of salpingitis in the patient with a mild clinical presentation must be confirmed.

Patients should be informed of the level of uncertainty in the clinical diagnosis of PID and should be given the option of more elaborate diagnostic evaluation. The label of "PID" as an STD should not be taken lightly by the clinician. This diagnosis often calls for a frank discussion between the patient and her sexual partner. Patients often choose to have more invasive testing when given the option in order to provide an accurate diagnosis. The clinician should provide the patient with the facts and allow the patient to play an active role in decisions concerning further diagnostic testing.

### CONCLUSIONS

The accurate diagnosis of PID is difficult, and the ramifications of misdiagnosis may be quite serious. A thorough evaluation for PID will include a careful history and physical examination with attention to evidence of lower genital tract infection based on inspection and vaginal wet preparation. Supportive laboratory evaluation may include an elevated ESR, CRP, and positive cultures from the lower genital tract. When numerous positive criteria are noted, the diagnostic specificity is high and further supportive evaluation may not be necessary.

Missing the diagnosis of PID (false negative) usually has more detrimental results than a false-positive diagnosis. Therefore, when PID is suspected, prompt treatment should be initiated as sensitivity is paramount. When additional specificity is sought (fewer false positives), as in severe cases when serious morbidity may result from misdiagnosis or when misdiagnosis results in considerable psychologic burden to the patient, then more elaborate supportive criteria such as an endometrial biopsy or a laparoscopy should be considered.

### REFERENCES

1. Wolner-Hanssen P, Kiviat NB, Holmes KK: Atypical pelvic inflammatory disease: Subacute, chronic, or subclinical upper genital tract infection in women. In Holmes KK, Mardh P-A, Sparling PF, Wiesner PJ, Cates W, Lemon SM, Stamm WE (eds): Sexually Transmitted Diseases. 2nd ed. New York: McGraw-Hill, pp 615–620, 1990.
2. Sweet RL, Mills J, Hadley KW, et al.: Use of laparoscopy to determine the microbiologic etiology of acute salpingitis. Am J Obstet Gynecol 134:68–74, 1979.
3. Jacobson L, Westrom L: Objectivized diagnosis of acute pelvic inflammatory disease: Diagnostic and prognostic value of routine laparoscopy. Am J Obstet Gynecol 105: 1088–1098, 1969.
4. Chaparro MV, Ghosh A, Nashed A, Poliak A: Laparoscopy for the confirmation and prognostic evaluation of pelvic inflammatory disease. Int J Gynaecol Obstet 15: 307–309, 1978.
5. Patton DL, Moore DE, Spadoni LR, et al.: A comparison of the fallopian tube's response to overt and silent salpingitis. Obstet Gynecol 73:622–630, 1989.
6. Alder MW, Belsey EH, O'Connor BH: Morbidity associated with pelvic inflammatory disease. Br J Vener Dis 58:151–157, 1982.
7. Sellors JW, Mahoney JB, Chernesky MA, Rath DJ: Tubal factor infertility: An association with primary chlamydial infection and asymptomatic salpingitis. Fertil Steril 49:451–457, 1988.
8. Hillis SD, Riduian J, Marchbanks PA, Wasserheit JN, Cates W, Westrom L: Delayed care of pelvic inflammatory disease as a risk factor for impaired fertility. Am J Obstet Gynecol 168:1503–1509, 1993.
9. Mardh P-A, Westrom L: Acute pelvic inflammatory disease. In Holmes KK, Mardh P-A, Sparling PF, Wiesner PJ, Cates W, Lemon SM, Stamm WE (eds): Sexually Transmitted Diseases. 2nd ed. New York: McGraw-Hill, pp 593–613, 1990.
10. McCormick WM: Pelvic inflammatory disease. N Engl J Med 330:115–119, 1994.
11. Peipert JF, Sweeney PJ: Diagnostic testing in obstetrics and gynecology: A clinician's guide. Obstet Gynecol 82: 619–623, 1993.
12. Kahn JG, Walker C, Washington AE, Landers DV, Sweet R: Diagnosing pelvic inflammatory disease. A comprehensive analysis and consideration for developing a new model. JAMA 266:2594–2604, 1991.
13. Washington AE, Aral SO, Wolner-Hanssen P, Grimes DA, Holmes KK: Assessing risk for pelvic inflammatory disease and its sequelae. JAMA 266:2581–2586, 1991.
14. Wolner-Hanssen P, Eschenbach DA, Paavonen J, et al.: Association between vaginal douching and acute pelvic inflammatory disease. JAMA 263:1936–1941, 1990.
15. Stone KM, Grimes DA, Magder L: Personal protection against sexually transmitted diseases. Am J Obstet Gynecol 155:180–188, 1986.
16. Lee NC, Rubin GL, Ory HW, et al.: Type of intrauterine device and the risk of pelvic inflammatory disease. Obstet Gynecol 62:1–6, 1983.
17. Vessey MP, Yeates D, Flavel R, et al.: Pelvic inflammatory disease and the intrauterine device: Findings in a large cohort study. Br Med J 282:855–857, 1981.
18. Kessel E: Pelvic inflammatory disease with intrauterine device use: A reassessment. Fertil Steril 51:1–11, 1989.
19. Lee NC, Rubin GL, Boruck R: The intrauterine device and pelvic inflammatory disease revisited: New results from the Women's Health Study. Obstet Gynecol 72: 1–6, 1988.
20. Peterson HB, Galaid EI, Cates W: Pelvic inflammatory disease. Med Clin North Am 74:1603–1615, 1990.

21. Senanayake P, Kramer DG: Contraception and the etiology of pelvic inflammatory disease: New perspectives. Am J Obstet Gynecol 138:852–860, 1980.
22. Svensson L, Mardh P-A, Westrom L: Infertility after acute salpingitis with special reference to *Chlamydia trachomatis*. Fertil Steril 40:322–329, 1985.
23. Wolner-Hanssen P, Eschenbach DA, Paavonen J, et al.: Decreased risk of symptomatic chlamydial pelvic inflammatory disease associated with oral contraceptive use. JAMA 263:54–59, 1990.
24. Svensson L, Westrom L, Mardh P-A: Contraceptives and acute salpingitis. JAMA 251:2553–2555, 1984.
25. Maslow AS, Davis CH, Choong J, et al.: Estrogen enhances attachment of *Chlamydia trachomatis* to human endometrial epithelial cells in vitro. Am J Obstet Gynecol 159:1006–1014, 1988.
26. Barron AI, Pasley JN, Rank RG, et al.: Chlamydial salpingitis in female guinea pigs receiving oral contraceptives. Sex Transm Dis 15:169–173, 1988.
27. Rank RG, White HJ, Hough AJ, et al.: Effect of estradiol on chlamydial genital infection of female guinea pigs. Infect Immun 38:699–705, 1982.
28. Rank RG, Barron AL: Specific effects of estradiol on genital mucosal antibody response in chlamydial ocular and genital infections. Infect Immun 55:2317–2319, 1987.
29. Pasley JN, Rank RG, Hough AJ, et al.: Absence of progesterone effects on chlamydial genital infection in female guinea pigs. Sex Transm Dis 12:155–158, 1985.
30. Paavonen J, Westrom LV: Diagnosis of pelvic inflammatory disease. In Berger GS, Weström LV (eds): Pelvic Inflammatory Disease. New York: Raven Press, pp 48–78, 1992.
31. Sweet RL, Blankfort-Doyle M, Robbie MO, Schachter J: The occurrence of chlamydial and gonococcal salpingitis during the menstrual cycle. JAMA 255:2062–2064, 1986.
32. Paavonen J, Kiviat N, Brunham RC, et al.: Prevalence and manifestations of endometritis among women with cervicitis. Am J Obstet Gynecol 152:280–286, 1985.
33. Westrom L: Diagnosis, Aetiology, and Prognosis of Acute Salpingitis. Thesis. University of Lund, Lund, Sweden, Studentlitteratur AB, 1976.
34. Svensson L, Westrom L, Ripa KT, et al.: Differences in some clinical and laboratory parameters in acute salpingitis related to culture and serologic findings. Am J Obstet Gynecol 138:1017–1021, 1980.
35. Hagdu A, Westrom L, Brooks C, et al.: Multivariate analysis of prognostic variables in patients with acute pelvic inflammatory disease. Am J Obstet Gynecol 155:954–960, 1986.
36. Paavonen J, Teisala K, Heinonen PK, et al.: Microbiological and histopathological findings in acute pelvic inflammatory disease. Br J Obstet Gynaecol 94:454–460, 1987.
37. Soper DE, Brockwell NJ, Dalton HP, Johnson D: Observations concerning the microbial etiology of acute salpingitis. Am J Obstet Gynecol 170:1008–1017, 1994.
38. Faro S, Martens M, Maccato M, Hammill H, Pearlman M: Vaginal flora and pelvic inflammatory disease. Am J Obstet Gynecol 169:470–474, 1993.
39. Brunham RC, Paavonen J, Stevens CE, et al.: Mucopurulent cervicitis: The ignored counterpart in women of urethritis in men. N Engl J Med 311:1–6, 1984.
40. Paavonen J, Critchlow C, DeRouen T, et al.: Etiology of cervical inflammation. Am J Obstet Gynecol 154:556–567, 1986.
41. Sellors J, Mahony J, Goldsmith C, Rath D, Mander R, Hunter B, Taylor C, Groves D, Richarson H, Chernesky M: The accuracy of clinical findings and laparoscopy in pelvic inflammatory disease. Am J Obstet Gynecol 164:113–120, 1991.
42. Jacobson L, Laurell C-B, Gennser G, Marholev K: Plasma protein changes induced by acute inflammation of the fallopian tubes. Int J Gynaecol Obstet 13:249–256, 1975.
43. Wasserheit JN, Bell TA, Kiviat NB, et al.: Microbial causes of proven pelvic inflammatory disease and efficacy of clindamycin and tobramycrin. Ann Intern Med 104: 187–193, 1986.
44. Lehtinen M, Laine S, Heinonen PK, et al.: Serum C-reactive protein determination in acute pelvic inflammatory disease. Am J Obstet Gynecol 154:158–159, 1986.
45. Hemila M, Henriksson L, Ylikorkala O: Serum CRP in the diagnosis and treatment of pelvic inflammatory disease. Arch Gynecol Obstet 241:177–182, 1987.
46. Paavonen J, Aine R, Teisala K, et al.: Chlamydial endometritis. J Clin Pathol 38:726–732, 1985.
47. Paavonen J, Aine R, Teisala K, Heinonen PK, Punnonen R: Comparison of endometrial biopsy and peritoneal fluid cytologic testing in the diagnosis of acute pelvic inflammatory disease. Am J Obstet Gynecol 151:645–650, 1985.
48. Timor-Tritsch IE, Rottem S (eds): Transvaginal Ultrasound. 2nd ed. New York: Elsevier, p 521, 1991.
49. Cacciatore B, Stenham U-H, Ylostalo P: Comparison of abdominal and vaginal sonography in the diagnosis of ectopic pregnancy. Obstet Gynecol 73:770–777, 1989.
50. Nasri MN, Setchell ME, Chard T: Vaginal scans in acute gynecology. Lancet 1:360–361, 1990.
51. Tessler FN, Perrella RR, Fleischer AC, Grant EG: Transvaginal sonographic diagnosis of dilated fallopian tubes. AJR 153:523–525, 1989.
52. Cacciatore B, Leminen A, Ingman-Friberg S, Ylostalo P, Paavonen J: Transvaginal sonographic findings in ambulatory patients with suspected pelvic inflammatory disease. Obstet Gynecol 80:912–916, 1992.
53. Mardh P-A: An overview of infectious agents in salpingitis, their biology, and recent advances in methods of detection. Am J Obstet Gynecol 138:933, 1980.
54. Eschenbach DA, Buchanan TM, Pollock HM, et al. Polymicrobial etiology of acute pelvic inflammatory disease. N Engl J Med 293:166, 1975.
55. Kiviat NB, Wolner-Hanssen P, Peterson M, et al.: Localization of *Chlamydia trachomatis* infection by indirect immunofluorescence and culture in pelvic inflammatory disease. Am J Obstet Gynecol 154:865–873, 1986.

56. Bowie WR, Jones H: Acute pelvic inflammatory disease in outpatients: Association with *Chlamydia trachomatis* and *Neisseria gonorrhoeae*. Ann Intern Med 95:685–688, 1981.
57. Wolner-Hanssen P, Paavonen J, Kiviat N, Young M, Eschenbach DA, Holmes KK: Outpatient treatment of pelvic inflammatory disease. Obstet Gynecol 71:595–600, 1988.
58. Loeffelholz MJ, Lewinski CA, Silver SR, Purohit AP, Herman SA, Buonagurio DA, Dragon EA: Detection of *Chlamydia trachomatis* in endocervical specimens by polymerase chain reaction. J Clin Microbiol 30:2847–2851, 1992.
59. Bass CA, Jungkind DL, Silverman NS, Bondi JM: Clinical evaluation of a new polymerase chain reaction assay for detection of *Chlamydia trachomatis* in endocervical specimens. J Clin Microbiol 2648–2653, 1993.
60. Witkin SS, Jeremias J, Toth M, Ledger WJ: Detection of *Chlamydia trachomatis* by the polymerase chain reaction in the cervices of women with acute salpingitis. Am J Obstet Gynecol 168:1438–1442, 1993.
61. Kiviat NB, Wolner-Hanssen P, Eschenbach DA, Wasserheit JN, Paavonen J, Bell RA, et al.: Endometrial histopathology in patients with culture-proved upper genital tract infection and laparoscopically diagnosed acute salpingitis. Am J Surg Pathol 14:167–175, 1990.
62. Swayne LC, Love MB, Karasick SR: Pelvic inflammatory disease: Sonographic-pathologic correlation. Radiology 151:751–755, 1984.
63. Berland LL, Lawson TL, Foley DW, Albarelli JN: Ultrasound evaluation of pelvic infections. Radiol Clin North Am 20:367–382, 1982.
64. Golden N, Cohen H, Gennari G, Neuhoff S: The use of pelvic ultrasonography in the evaluation of adolescents with pelvic inflammatory disease. Am J Dis Child 141: 1235–1238, 1987.
65. Hager WD, Eschenbach DA, Spence MR, Sweet RL: Criteria for diagnosis and grading of salpingitis. Obstet Gynecol 61:113–114, 1983.
66. Sweet RL: Pelvic inflammatory disease and infertility in women. Infect Dis Clin North Am 1:209, 1987.
67. Soper DE: Diagnosis and laparoscopic grading of acute salpingitis. Am J Obstet Gynecol 164:1370–1376, 1991.
68. CDC: 1993 Sexually transmitted diseases treatment guidelines. MMWR 42(RR-14):75–81, 1993.
69. Binstock M, Muzsnai D, Apodaca L, et al.: Laparoscopy in the diagnosis and treatment of pelvic inflammatory disease: A review and discussion. Int J Fertil 31:341, 1986.
70. Burchell HJ, Schoon MG: The value of laparoscopy in the diagnosis of acute pelvic inflammatory disease. S Afr Med J 72:197, 1987.
71. Cates W Jr, Rolfs TRT, Aral SO: Sexually transmitted diseases, pelvic inflammatory disease and infertility: An epidemiologic update. Epidemiol Rev 12:199–220, 1990.
72. Moore DE, Cates W Jr: Sexually transmitted diseases and infertility. In Holmes KK, Mardh P-A, Sparling PF, et al. (eds): Sexually Transmitted Diseases. 2nd ed. New York: McGraw-Hill, pp 763–769, 1990.
73. Cates W Jr, Wasserheit JN: Genital chlamydial infections: Epidemiology and reproductive sequelae. Am J Obstet Gynecol 164:1771–1781, 1991.
74. Cates W Jr, Joesoef R, Goldman MB: Atypical pelvic inflammatory disease: Can we identify clinical predictors? Am J Obstet Gynecol 169:341–346, 1993.
